# Longitudinal deep learning-based segmentation of multiple sclerosis lesions in a local clinical cohort

**DOI:** 10.3389/fradi.2026.1758276

**Published:** 2026-08-12

**Authors:** Amalie Monberg Hindsholm, Annika Reynberg Langkilde, Jette Lautrup Frederiksen, Flemming Littrup Andersen, Henrik B. W. Larsson, Ulrich Lindberg, Claes Nøhr Ladefoged

**Affiliations:** 1 Department of Clinical Physiology and Nuclear Medicine, Copenhagen University Hospital -Rigshospitalet, Copenhagen, Denmark; 2 Department of Radiology, Copenhagen University Hospital - Rigshospitalet, Copenhagen, Denmark; 3 Department of Neurology, Danish Multiple Sclerosis Center, Copenhagen University Hospital -Rigshospitalet, Glostrup, Denmark; 4 Department of Clinical Medicine, University of Copenhagen, Copenhagen, Denmark; 5 Department of Applied Mathematics and Computer Science, Technical University of Denmark, Kongens Lyngby, Denmark

**Keywords:** deep learning, lesion segmentation & quantification, longitudinal MRI, multiple sclerosis, new lesion detection, nnU-Net

## Abstract

**Purpose:**

Automated monitoring of multiple sclerosis (MS) lesion progression remains challenging in clinical practice. This study evaluates LongiSeg, a longitudinal deep learning architecture, on heterogeneous clinical data to assess its practical value for routine MS radiological monitoring and extends its use for new lesion detection.

**Methods:**

We trained LongiSeg on 470 patients from a diverse clinical cohort acquired across 15 scanner types (both 1.5T and 3T) with mixed 2D/3D T2w FLAIR sequences. We trained and evaluated models for both cross-sectional lesion segmentation and new lesion detection. Performance was compared against a single-timepoint nnU-Net on a cross-sectional test cohort consisting of 67 patients as well as 22 patients presenting with new lesions.

**Results:**

LongiSeg achieved superior cross-sectional segmentation performance compared to single-timepoint nnU-Net on the clinical test set (*n* = 67, T2w FLAIR input, DSC: 0.684 ± 0.124 vs. DSC: 0.665 ± 0.144). LongiSeg for new lesion detection obtained a low performance on the small clinical test cohort (*n* = 22, DSC: 0.260, 95%CI: 0.143–0.383).

**Conclusion:**

LongiSeg demonstrated moderate improvements in cross-sectional MS lesion segmentation. However, the added complexity of processing longitudinal scans may not be justified by these modest gains. For new lesion segmentation, performance was low, especially in cases with few lesions.

## 1 Introduction

Multiple sclerosis (MS) is a chronic, inflammatory disease of the central nervous system (CNS) affecting millions of people worldwide (1). The disease is characterised by episodes of partially reversible neurological disability, associated with the formation of demyelinating lesions in the CNS (2). Magnetic Resonance Imaging (MRI) visualisation of these lesion patterns serves as a critical biomarker for disease progression and treatment response. Following MAGNIMS-CMSC-NAIMS consensus recommendations, patients undergo regular MRI examinations at 6–12-month intervals after diagnosis and 3–6 months after treatment initiation (3). Neuroradiologists assess disease activity by comparing current MRI scans to previous time points, tracking overall lesion burden and identifying new and enlarged lesions (4). This process is labour-intensive and subject to inter- and intra-rater variability, motivating extensive research into automated MS lesion analysis.

Initial efforts in automated MS assessment focused on cross-sectional lesion segmentation, the delineation of all lesions visible on a single scan, from single-timepoint MRI. Deep learning approaches have achieved near-human performance on this task (5, 6), with the U-Net architecture (7) inspiring several high-performing segmentation models (5, 8–15). Comprehensive overviews of deep learning methods for MS detection and analysis are provided in Belwal and Singh (16) and Thabet et al. (17). Recently, several single-timepoint approaches built on the self-configuring nnU-Net framework (18) have been successfully applied to MS lesion segmentation (19–21). Cross-sectional segmentation enables subsequent longitudinal comparison by spatially registering follow-up scans to the baseline and identifying overlapping lesions—thereby facilitating lesion-level tracking and quantification of disease progression, both of which are critical for clinical assessment of disease activity.

However, single-timepoint models do not utilise the complementary temporal information available across consecutive scans, which can reduce sensitivity to subtle changes and limit robustness. Therefore, longitudinal models have emerged for cross-sectional lesion segmentation. These are defined as models accepting MRI from multiple consecutive time-points as input for each case, providing the model with information of temporal changes. The convolutional neural network (CNN) has been proposed for several longitudinal models, with different approaches to the fusion of spatial representations (22, 23) and solutions to data scarcity (24).

Though models for cross-sectional lesion segmentation have achieved a high segmentation performance, they are dependent on subsequent lesion tracking to provide information on new and enlarged lesions, introducing several drawbacks. First, lesion tracking based on spatial overlap can fail due to imperfect registration, lesion evolution, or segmentation inconsistencies, which may lead to misclassification or missed detections. Second, this approach does not reflect the clinical decision-making process, where radiologists assess progression by jointly reviewing both scans and integrating anatomical context, visual cues, and expert judgement that is not captured by automated overlap-based strategies (3).

In contrast to cross-sectional segmentation approaches, a distinct group of methods has emerged that directly target the identification of disease progression—specifically, the detection and segmentation of new lesions across timepoints. These approaches avoid *post-hoc* overlap-based challenges by learning temporal patterns and changes directly, closely emulating the clinical workflow. However, these methods often face several other challenges such as class imbalance and limited access to labelled datasets (25). Several proposed methods utilise the U-Net architecture, reading consecutive MRIs as separate input channels, combined with reference masks with only new lesions delineated. Model variations have included different approaches to feature learning, including a dual-path encoder for initial individual feature learning (26), learning a feature deformation field through cascaded U-Nets (27), and a triplanar U-Net to learn common features across orientations (28). Other papers have focused on tackling the extensive class imbalance and data scarcity often present in datasets for new lesion segmentation, through oversampling strategies (29, 30) or GAN-based preprocessing pipelines for denoising and data augmentation (31).

nnU-Net has also been proposed for new lesion segmentation, with Martinez-Heraz et al. (32) employing a standard nnU-Net with a two-channel input, while Basaran et al. (33) included additional preprocessing and data augmentation steps to generate diverse data samples and tackle data scarcity. While some models have addressed lesional changes (26, 27, 34), covering both new and enlarged lesions, most of the community's efforts, including most proposed models and benchmark challenges such as the 2021 MICCAI new lesion segmentation challenge (35), have concentrated primarily on segmenting new lesions.

Recently, Rokuss et al. (36) introduced LongiSeg, a longitudinal nnU-Net adaptation for cross-sectional lesion segmentation incorporating architectural inductive bias through difference weighting blocks. LongiSeg was evaluated on the ISBI 2015 (6) dataset and demonstrated superior performance compared to other longitudinal models for cross-sectional lesion segmentation. They did, however, note that state-of-the-art single-timepoint models remained competitive, highlighting the need for better benchmarking of longitudinal approaches.

In this study, we trained and evaluated LongiSeg for two clinically relevant tasks using a diverse local clinical dataset. First, we assessed cross-sectional MS lesion segmentation, comparing LongiSeg against a standard nnU-Net baseline and evaluating generalisability on two public benchmark datasets [Ljubljana (37) and ISBI (6, 38)]. Second, we evaluated LongiSeg's difference-weighting design for new lesion segmentation on a sub-cohort of patients with confirmed new lesions, again benchmarking against a public dataset [MSSEG-2 (35)]. Across both tasks, we assessed the practical value of LongiSeg's longitudinal design for routine clinical MS monitoring, using a real-world heterogeneous clinical dataset.

## 2 Methods

### 2.1 Data

In this study we utilised a large, local clinical MS MRI dataset as well as three public datasets for benchmarking. The clinical dataset was used as our main dataset for model training and testing throughout.

#### 2.1.1 Clinical datasets

The clinical dataset was comprised of two local datasets: a primary dataset used for model development and validation of cross-sectional segmentation performance, and a secondary dataset that was combined with the primary dataset, specifically to evaluate segmentation of new lesions only. The primary dataset is a subset of an original cohort consisting of a single-timepoint examinations from 746 patients who were referred for routine MS MRI examination between January 2015 and October 2020 at our local MS clinic (21). The cohort was originally used to develop and validate our MS lesion delineation method, AI for MS (*AIMS)*^1^ (21). Of this cohort we included all patients (*n* = 537) who had an additional, prior examination in our database enabling longitudinal analysis. The prior examinations were, on average, acquired 14.5 (range: 6–30) months before the original MRI. Demographic and image protocol details are provided in Supplementary Table S1. We used the T2-weighted (T2w) and T2w FLAIR images in this study. The secondary dataset consisted of patients with new lesion segmentations from a recent prospective validation study (*n* = 13) (39). A summary of the dataset's lesion statistics including total, median and IQR can be found in Table 1.

**Table 1:** Summarised lesion statistics for each of the two lesion types: cross-sectional and new, based on their respective reference masks.

Cross-sectional lesion statistics	
Patients (*n*)	537
Total lesions (*n*)	23,481
Lesion count at follow-up, median [IQR] (range)	35 [19–56] (2–280)
lesion volume at follow-up (mm^3^), median [IQR] (range)	4,639.1 [1,831.3–11,226.2] (21.7–66,003.0)

New lesion statistics	
Patients (*n*)	87
Total lesions (*n*)	301
Lesion count at follow-up, median [IQR] (range)	2 [1–4] (1–10)
lesion volume at follow-up (mm^3^), median [IQR] (range)	113.5 [41.3–232.7] (0.01–1,769.0)

The cross-sectional lesions were estimated on the complete clinical cohort. The new lesions were estimated on the sub-set of the clinical cohort, which had a minimum of 1 new lesion. In both instances, reference masks were only available on the follow-up scans, and lesion statistics are therefore only reported for these.

#### 2.1.2 Public datasets

1. ISBI 2015 Longitudinal MS Lesion Segmentation Challenge (6, 38) This dataset includes five patients with a total of 21 timepoints (four patients with four timepoints each, and one with five), acquired at a single scanner. Each time point contains a T1w magnetization prepared rapid gradient echo (MPRAGE), proton density (PD) weighted, T2w, and T2w FLAIR image. We only used the T2w FLAIR and T1w images and the associated cross-sectional lesion segmentations. No annotations for new or enlarging lesions are available.
2. Ljubljana Dataset (37) Provided by the University Medical Center Ljubljana and the Laboratory of Imaging Technologies, University of Ljubljana, this dataset comprises 162 patients with one to four timepoints each, totaling 331 examinations. Of these, 30 cases are publicly accessible, while the remainder can be requested. Each timepoint contains a 3D T2w FLAIR, a 2D T1w turbo inversion recovery magnitude (TIRM) image, and a cross-sectional lesion segmentation derived from consensus among three readers, acquired at a single scanner. No classification of new or enlarging lesions is included.
3. MSSEG-2 (MICCAI 2021 New Lesion Segmentation Challenge) (35) This dataset contains 40 patients, each with two timepoints, acquired at a total of 12 scanner models. 29 of 40 had at least one new lesion between first and second timepoint. All timepoints include 3D T2w FLAIR images and annotations of new lesions.

Image protocol details are provided for the public datasets in Supplementary Data.

#### 2.1.3 Data splits

From the primary clinical dataset, we retained the original train/test split from (21) for our clinical cohort, using 470 patients for training (65 with new lesions) and 67 for testing (9 with new lesions). The secondary dataset of 13 patients was only used as part of the test dataset for segmentation of new lesions and was combined with the primary dataset. This resulted in a final test-cohort for new lesion segmentation of 22 patients. The train/test splits of our clinical dataset were kept throughout the study, to avoid data leakage. The Ljubljana dataset was used for both training and evaluation, following the split described in (36): 129 patients (265 scans) for training and 33 patients (66 scans) for testing. The ISBI dataset was used exclusively for benchmarking against other cross-sectional methods and was therefore included only for testing. The MSSEG-2 dataset was used either for training (the 29 with new lesions) or benchmark testing, depending on the experiment (see Sec. 2.5.3). An overview of the datasets and their training/test splits are presented in Table 2.

**Table 2:** Overview of the four datasets and their splits into train/test sets at patient level. Total number of scans per split shown in parentheses.

Dataset	Modalities included	Cross-sectional		New lesion	
		Train	Test	Train	Test
Clinical	T2w FLAIR & T2w	470 (944)	67 (134)	65	22
ISBI	T2w FLAIR & T1w	—	5 (21)	N/A	N/A
Ljubljana	T2w FLAIR & T1w	129 (265)	33 (66)	N/A	N/A
MSSEG-2	T2w FLAIR	N/A	N/A	40*	40*

* The MSSEG-2 patients were used either for training or testing depending on the experiment. Of the 40 cases, 29 had new lesions.

#### 2.1.4 Clinical datasets, lesion masks

**Figure 1:**
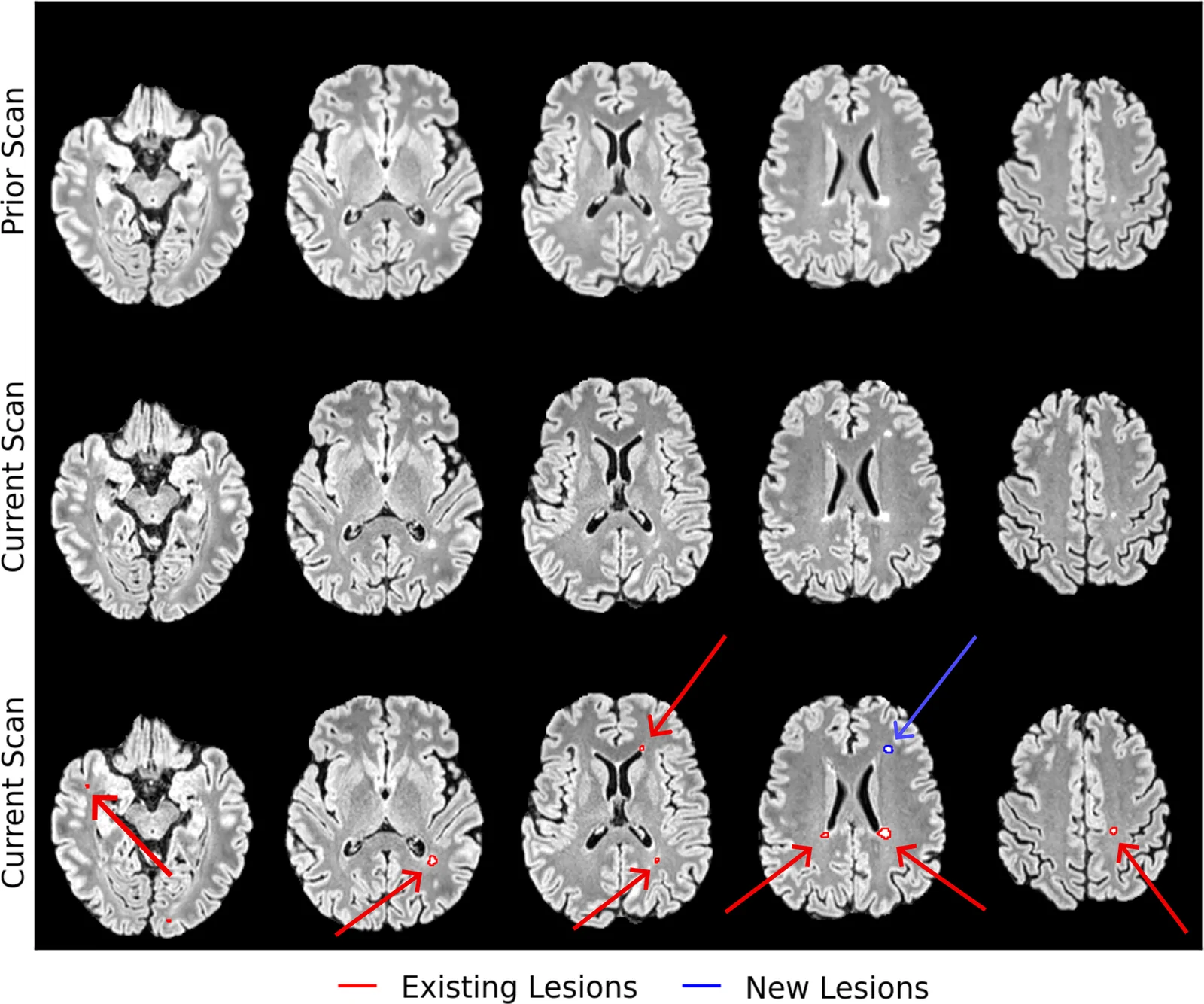
An example of the two types of delineations on an example patient from the clinical test dataset: all lesion segmentation (red) and new lesion segmentation (blue).

Reference lesion masks were available for the current timepoints in the clinical cohort (*n* = 21) and served as the cross-sectional ground truth. For the cohort also used for new-lesion segmentation (*n* = 65/22 for train/test, Table 2), lesions were further classified as stationary or new through a two-step process. First, the prior scan was segmented using AIMS, and candidate new lesions were defined as those present in the current scan with no overlap in the prior scan. Second, the number of new lesions was cross-referenced against the Danish Multiple Sclerosis Registry (DMSR) (40), which routinely records the number of new lesions at each follow-up examination. Classifications were manually reviewed and adjusted until the number of new lesions matched the registry; lesions from the original mask not classified as new were labeled stationary. This review was performed by a trained engineer with four years of experience in MS lesion delineation for research purposes, trained by an experienced neuroradiologist. Figure 1 shows an example of new- and all-lesion reference delineations for a single patient from the test dataset. The combined new-lesion test dataset (*n* = 22) was independently validated by a neuroradiologist with more than 20 years of experience, who reviewed and corrected the new-lesion delineations generated by an automatic tool. Only newly appearing lesions were included; enlarged or reduced lesions were not considered.

### 2.2 Preprocessing and data harmonisation

#### 2.2.1 Clinical dataset

Initial preprocessing of the clinical dataset followed (41), and consisted of the following steps: (1) All inputs were reoriented to standard orientation [fslreorient2std (42, 43)], (2) Brain extraction was performed on all MRI sequences [HD-BET (44)], (3) T2w images were linearly registered to T2w FLAIR space using rigid transformation with 6 degrees of freedom [FSL FLIRT version 5.0 (42, 43)], (4) Prior T2w FLAIR images were registered to current T2w FLAIR space with a rigid transformation (6 dof) [FreeSurfer v 7.4.1, mri_robust_register (45)]. Nearest neighbour interpolation was used when resampling segmentation masks, while cube interpolation was applied for image resampling (5), For each prior T2w image, the registration matrices from registration to prior T2w FLAIR space and from prior to current T2w FLAIR space were concatenated and applied [FreeSurfer lta_convert (42, 43)].

#### 2.2.2 Public dataset

All public datasets used in this study were pre-processed prior to retrieval. For the Ljubljana and ISBI datasets, preprocessing included alignment of images to a common anatomical space, correction for intensity inhomogeneity, and skull-stripping. The MSSEG-2 dataset was provided with aligned images only. To ensure consistency with the other datasets, we applied skull-stripping using HD-BET.

### 2.3 Ethics statement

All patient-specific data was handled in compliance with the Danish data protection agency act no. 502. Collection of the clinical retrospective dataset was approved by the National Committee on Health Research Ethics (protocol number 2117506). All patient-specific data from the local dataset were pseudo-anonymised and the ISBI and Ljubljana datasets were fully anonymised before we retrieved them. The GDPR (the European General Data Protection Regulation) was thereby fulfilled.

### 2.4 Networks

#### 2.4.1 nnU-Net

The nnU-Net (18) is a deep learning-based segmentation framework, comprised of pre-processing, training, validation and inference. The deep learning network itself builds on a classical U-net architecture, employing skip connections between the encoding and decoding paths (7). The framework extracts a set of fingerprints from the dataset, which automatically adapts several hyperparameters in the training setup, including patch- and batch size. Extensive data augmentation is employed, including rotations, scaling, gaussian noise, brightness and contrast. Validation is by default done by training a 5-fold cross-validation scheme. The default nnU-Net training scheme further includes stochastic gradient descent optimiser with momentum 0.99, an initial learning rate of 0.01 and polynomial decay.

#### 2.4.2 Longitudinal nnU-Net, LongiSeg

LongiSeg (46) extends the nnU-Net framework to incorporate scans from multiple time-points. It offers two fusion strategies, which we will label LongiSeg and LongiSegDW respectively: 1) LongiSeg includes a simple channel wise concatenation of the prior and current images before input to the network, which is a standard nnU-Net, 2) LongiSegDW includes a more advanced approach where the two time points are encoded separately and fused during decoding using difference weighting (DW) blocks applied to all skip connections. These blocks compute the element-wise difference between the prior and current feature maps, apply instance normalization, multiply the result with the current features, and finally add a residual connection to preserve original information and enhance temporal integration. We refer to the original publication for further details on the architecture and DW blocks (46).

During training, both LongiSeg and LongiSegDW randomly selects one of the available scans as the current time point, with the prior scan defined as the one immediately preceding it. Since detailed lesion segmentation masks were only available for the most recent scans in our dataset, we created a new data loader to be used in the in the LongiSeg and LongiSegDW framework, that uses a fixed current scan—always selecting the latest available scan as the current input.

### 2.5 Experiments

#### 2.5.1 Training parameters

As LongiSeg is based on the nnU-Net, there are several overlaps between the training parameters for both models. Models were implemented using nnU-Net-v2 in the 3D full-resolution configuration with residual connections in the encoder, automatically adapted to a large-size VRAM budget (nnU-Net ResEncL) (47). All models were trained for 1,000 epochs, training only 1 of five folds of the default cross-validation. The 1-fold cross-validation split was then used to create a single train-and validation split within the training dataset cohort. Patch and batch sizes were auto-configured per dataset utilising the automatic fingerprint extraction of nnU-Net (18) and therefore differ slightly across models. All models shared six encoder stages with feature maps [32, 64, 128, 256, 320, 320], instance normalisation, and LeakyReLU activations. Key configuration parameters for all models are summarised in Supplementary Tables S3, S4. Through analysis of loss curves from all models, it was decided to us the *best* model checkpoint instead of the *final*, as is default by nnU-Net. This was done to minimise overfitting on especially the new lesion datasets. All loss curves can be found in Supplementary Figures S1–9. This analysis was done using the validation datasets from the first fold of internal cross-validation splits by nnU-Net, and not the clinical test dataset.

#### 2.5.2 Cross-sectional lesion segmentation

Three cross-sectional lesion segmentation models were trained on the clinical training split (*n* = 470, Table 2): nnU-Net taking a single-timepoint input (i.e., current MRI only), LongiSeg taking a longitudinal data input (prior and current MRI) with channel-wise concatenation, and LongiSegDW using the same longitudinal input with DW blocks. All models were initially given T2w FLAIR images as input. Additionally, nnU-Net and LongiSegDW were retrained using both T2w and T2w FLAIR images.

Models trained on the clinical dataset were evaluated on the clinical test set (*n* = 67). The LongiSegDW model was further evaluated on both public test datasets Ljubljana and ISBI to establish benchmarking performance. To enable direct comparison with Rokuss et al. (36), a LongiSegDW model was trained on the Ljubljana training split (*n* = 129, Table 2) using T2w FLAIR and T1w input. The Ljubljana-trained LongiSegDW was evaluated on the Ljubljana and ISBI test sets only. To assess whether acquisition dimensionality mismatch between prior and current T2w FLAIR images introduced a systematic bias in longitudinal model performance, the results from LongiSegDW on the clinical test set were stratified by acquisition pairing (2D → 2D, 3D → 3D, and mixed).

#### 2.5.3 New lesion segmentation

Three LongiSegDW models were trained for new lesion segmentation. Each model took a single-sequence input of T2w FLAIR images from three different training cohorts: (1) the local clinical cohort (*n* = 65, Table 2), (2) the public MSSEG-2 cohort containing new lesions (*n* = 29), and (3) a combined dataset that included both cohorts (*n* = 94). All models were trained using binary lesion masks as reference, where voxels corresponding to new lesions were labelled as 1 and all others as 0 (background). The models were evaluated on the new lesion sub-cohort of our clinical test set. Model (1) was further benchmarked on the MSSEG-2 dataset.

#### 2.5.4 Statistical testing

Statistical comparisons between models evaluated on the same test cohort were performed using the paired Wilcoxon signed-rank test on per-subject DSC and F1-score values. The Wilcoxon signed-rank test was used for all pairwise comparisons to ensure a consistent non-parametric approach, as the distribution of per-subject metric differences could not be assumed to be normal across all comparisons. The two-sided Mann–Whitney *U*-test was calculated for comparison between three different groups, when stratifying cross-sectional lesion segmentation performance between matched and mismatched acquisition pairs. Where multiple pairwise comparisons were carried out within the same family of tests, a Bonferroni correction was applied to control the family-wise error rate. Bootstrap 95% confidence intervals were estimated by resampling subjects with replacement across 10,000 iterations. These were reported for the new lesion segmentation results due to the small test cohort size (*n* = 22) and for the stratified analysis by T2w FLAIR acquisition pairing.

### 2.6 Performance metrics

Model performance was evaluated by comparing reference and predicted segmentation masks. Individual lesions were identified from both reference and predicted segmentations using connected component analysis [skimage.measure.label(), scikit-image v0.24.0 (48)], applying the default 26-connectivity in 3D, where voxels sharing a face, edge, or corner are considered part of the same lesion. For new lesion segmentation, reference masks contained only new lesions generated independently of pre-existing lesion masks, meaning adjacency between new and pre-existing lesions did not affect connected component delineation in the reference. Model performance was evaluated using four metrics operating at two levels: voxel and lesion-wise. Lesion overlap was defined as any voxel overlap between the reference and predicted mask, and a minimum lesion volume cutoff of 3 mm^3^ was applied, as done by Rokuss et al. (36). At the voxel level, the Dice Similarity Coefficient (DSC) was used to quantify segmentation overlap between prediction and reference (36):

$$DSC=\frac{2|A\cap G|}{\left|A|+|G\right|}$$

Where G denotes the number of lesion voxels in the reference and A in the prediction. At the lesion level, precision (positive predictive value), recall (true positive rate), and F1-score assess detection performance:

$$\begin{array}{rl} P\mathrm{recision} & =\frac{\mathrm{TP}}{\mathrm{TP}+\mathrm{FP}},\mathrm{Recall}=\frac{\mathrm{TP}}{\mathrm{TP}+\mathrm{FN}},\\ F1 & =\frac{2\times \mathrm{Precision}\times \mathrm{Recall}}{\mathrm{Precision}+\mathrm{Recall}} \end{array}$$

where TP, FP, and FN denote true positive, false positive, and false negative lesion detections, respectively. Precision and recall are reported separately for transparency, as they jointly determine the F1-score. To allow for detailed comparison of lesion segmentations across studies, we additionally calculated voxel-wise precision, recall and F1-score, as well as the Pearson correlation coefficient between predicted and reference total lesion volumes per patient.

For the new lesion segmentation sub-study, lesion-wise metrics were calculated for all test subjects with annotated new lesions (*n* = 22). To assess patient-level classification performance, each patient was classified as active (≥1 new lesion voxel in the mask) or stable (empty mask), and classification metrics were computed on the combined cohort of active (*n* = 22) and stable (*n* = 58) patients, where the 58 stable patients were those from the test set without new lesions recorded in the DMSR. Patient-wise precision, recall, specificity, negative predictive value (NPV), F1-score, and balanced accuracy (mean of recall and specificity) were calculated from the resulting confusion matrix.

## 3 Results

### 3.1 Cross-sectional lesion segmentation

**Figure 2:**
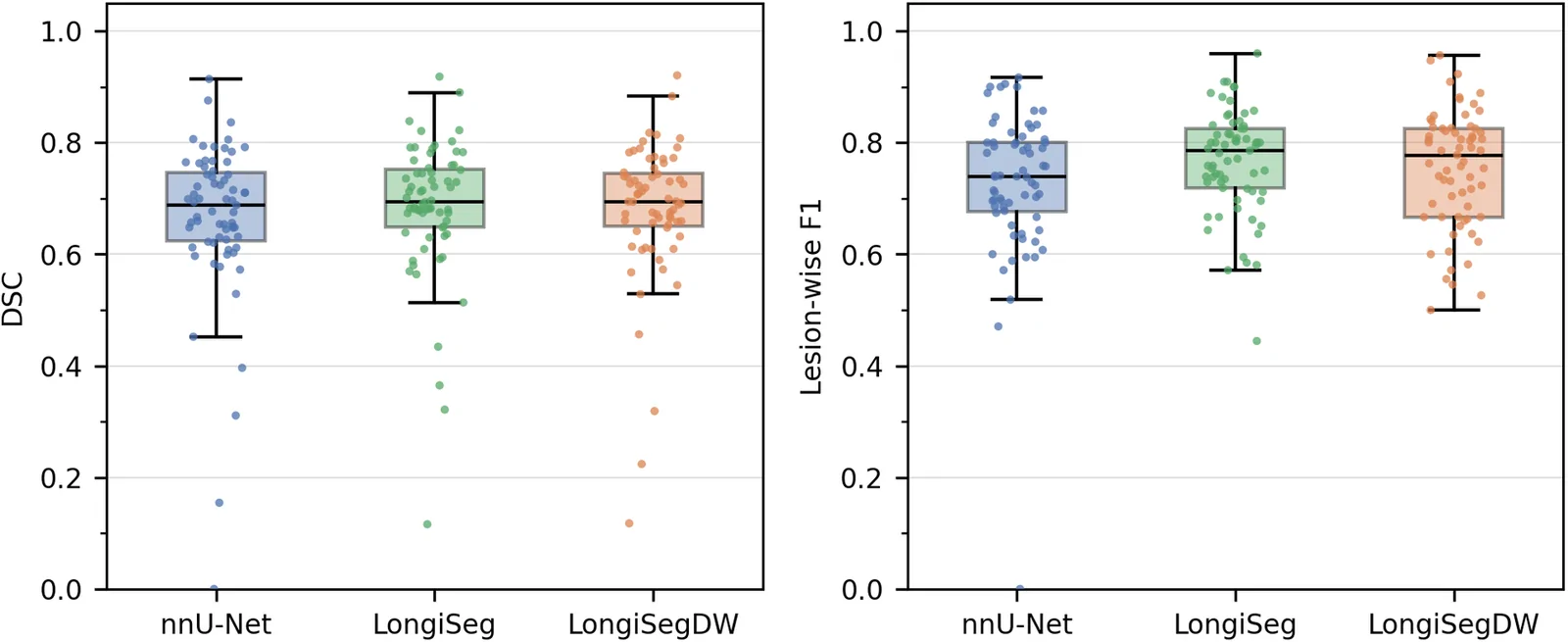
Distribution of DSC and lesion-wise F1-score for the three main models for cross-sectional lesion segmentation: nnU-Net, LongiSeg and LongiSegDW. All three models were trained taking a single-sequence input of T2w FLAIR images.

**Figure 3:**
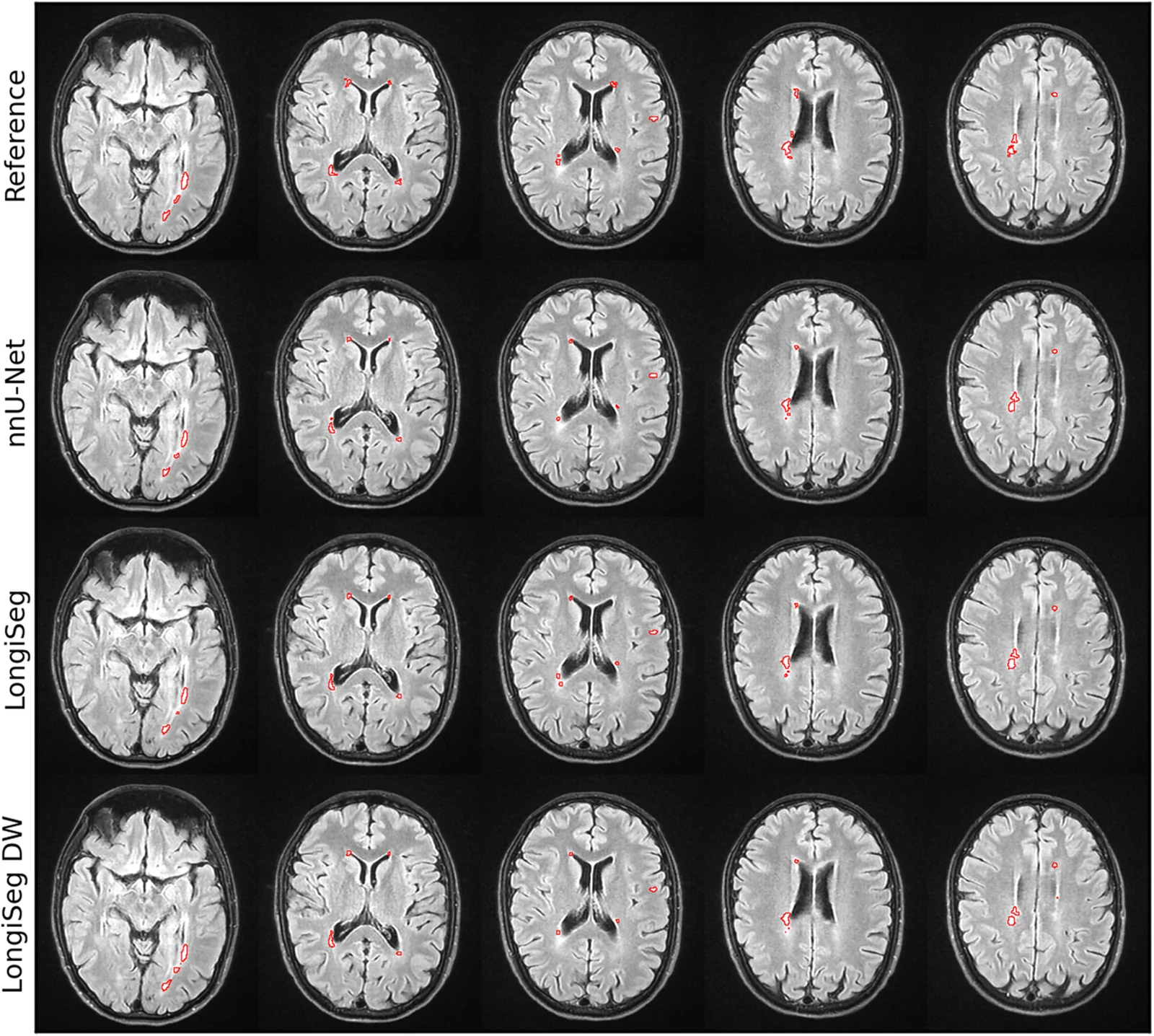
Example slices with delineations made by the single-input nnU-Net, LongiSeg and LongiSeg with difference weighting (DW), on a patient from the clinical test dataset. All three models are trained with T2w FLAIR input only. Though nnU-Net performed significantly worse on the average segmentation performance, there are minimal visible differences in the delineations on the example patient, indicating modest performance gains when changing to LongiSeg.

Cross-sectional lesion segmentation performance on the clinical test set is summarised in Table 3, with voxel-wise metrics in Supplementary Table S5. For the models trained with T2w FLAIR input only, both LongiSeg and LongiSegDW obtained higher average DSC and F1 than the single-timepoint nnU-Net model. They obtained mean DSC increases of 0.02 and mean F1 increases of 0.03 and 0.02 respectively—all statistically significant (DSC: *p* < 0.001, F1: *p* < 0.001)). LongiSeg and LongiSegDW differed significantly from each other regarding DSC (DSC: *p* = 0.009), but not F1-score (F1: *p* = 0.156). An example of delineations made by each model on a patient with median DSC on LongiSegDW can be found in Supplementary Figure S10. The distributions of DSC and F1-score across patients for all three models can be found in Figure 2. When T2w images were provided as additional input alongside T2w FLAIR, none of the models performed significantly different to their single-input performance (*p* > 0.05). LongiSegDW with two input channels did not perform significantly different from nnU-Net with two input channels with regards to DSC (DSC: *p* = 0.131) but did with regards to F1 (F1: *p* = 0.039). Full pairwise statistical comparisons are provided in Supplementary Tables S6, 7. Examples of delineations made by the three T2w FLAIR-only models are presented in Figure 3.

**Table 3:** Comparison of models for cross-sectional lesion segmentation trained and evaluated on our local clinical dataset. All models were trained and tested on the same patient cohorts (*n* = 470/67 for train/test).

Model name	Train dataset	DSC	F1-score	Precision	Recall
Models trained with T2w FLAIR input					
nnU-Net	Clinical, single timepoint	0.6648 (0.1438)	0.7259 (0.1319)	0.9009 (0.1486)	0.6267 (0.1569)
LongiSeg	Clinical, longitudinal	**0.6838** **(0.1243)**	**0.7661** **(0.0930)**	**0.9035** **(0.1012)**	**0.6848** **(0.1382)**
LongiSegDW	Clinical, longitudinal	0.6782 (0.1287)	0.7529 (0.1046)	0.9000 (0.1145)	0.6673 (0.1415)
Models trained with T2w FLAIR and T2w input					
nnU-Net	Clinical, single timepoint	0.6695 (0.1368)	0.7410 (0.1034)	0.8949 (0.1190)	0.6501 (0.1350)
LongiSegDW	Clinical, longitudinal	0.6792 (0.1253)	0.7619 (0.1152)	0.8722 (0.1193)	0.6986 (0.1493)

nnU-Net models were trained using MRI from a single timepoint from each patient, LongiSeg models were trained on images from two timepoints from each patient. The first models used T2w FLAIR input only, while the last two used both T2w FLAIR and T2w images. Values are means across subjects in each test cohort, with standard deviation reported in parenthesis. Bold indicates the highest average metric value across each metric—see main text and supplementary Tables for pairwise significance testing. DSC, dice similarity coefficient; DW: difference weighting.

Stratified analysis by T2w FLAIR acquisition pairing revealed comparable performance between matched 2D → 2D and mismatched pairs (DSC 0.704 vs. 0.699, Table 4), suggesting that within our clinical test set, acquisition mismatch did not significantly affect LongiSegDW performance. Calculating the two-sided Mann–Whitney *U*-test between the three groups, there were no significant changes between matched and mismatched groups. The complete table of results for the two-sided Mann–Whitney *U*-tests can be found in Supplementary Table S8.

**Table 4:** Performance of LongiSegDW with T2w FLAIR input, on the clinical test set (*n* = 67) stratified by FLAIR acquisition pairing between prior and current timepoints.

Pairing	*n*	DSC	F1	Precision	Recall
2D,2D	25	0.704 (0.672–0.733)	0.771 (0.730–0.808)	0.865 (0.815–0.904)	0.718 (0.664–0.769)
3D,3D	24	0.636 (0.559–0.701)	0.761 (0.716–0.803)	0.939 (0.891–0.975)	0.653 (0.598–0.707)
2D,3D	18	0.699 (0.662–0.736)	0.717 (0.675–0.761)	0.897 (0.845–0.941)	0.617 (0.555–0.681)

Values are means across subjects with 95% bootstrap confidence intervals in parentheses (10,000 iterations, sampling with replacement). 2D,3D indicates mismatched acquisition dimensionality between prior and current scan.

The 3D → 3D subgroup showed lower DSC (0.639) than the other two permutations, with wider confidence intervals. The difference was, however, not significant (*p* < 0.017 Bonferroni corrected threshold). Looking at the patient distributions of DSC and F1-score in Supplementary Figure S11, the low average DSC appears to be driven by three very low scores, corresponding to patients with very small new lesion volumes (Supplementary Figure S12), for which voxel-wise DSC is known to be unstable.

The LongiSegDW model trained and tested on the Ljubljana dataset significantly outperformed the model trained on our clinical dataset (DSC: *p* < 0.001, F1: *p* < 0.001). No statistically significant difference was observed between the two models on the ISBI test dataset (DSC *p* < 1.0, F1: 0.812); however, given the small size of this test set (*n* = 5), this comparison is likely underpowered to detect a true difference and should be interpreted with caution. The primary metrics are provided in Table 5, with full pairwise statistical comparisons provided in Supplementary Table S10 and additional woxel-wise metrics in Supplementary Table S9.

**Table 5:** Benchmark performance of LongiSegDW tested on public datasets for cross-sectional MS lesion delineation.

Model (input)	Train dataset	Test dataset	DSC	F1-score	Precision	Recall
LongiSegDW (T2w FLAIR & T1w)	Ljubljana (*n* = 129)	Ljubljana (*n* = 33)	**0.7568 (0.0806)**	**0.8130 (0.0603)**	0.8831 (0.0739)	**0.7637 (0.1001)**
LongiSegDW (T2w FLAIR)	Clinical (*n* = 470)	Ljubljana (*n* = 33)	0.6656 (0.1128)	0.7476 (0.0714)	**0.9159 (0.0720)**	0.6394 (0.0938)
LongiSegDW						
(T2w FLAIR & T1w)	Ljubljana (*n* = 129)	ISBI (*n* = 5)	0.7223 (0.0528)	**0.7181 (0.0867)**	0.7230 (0.0854)	0.7165 (0.0976)
LongiSegDW						
(T2w FLAIR)	Clinical	ISBI (*n* = 5)	**0.7242 (0.0542)**	0.7165 (0.1124)	0.9001 (0.0898)	0.6018 (0.1284)

The number in the dataset column parentheses indicates the number of patients with consecutive scans. When trained on the Ljubljana dataset, T1w images were included as input. Values are means across subjects in each test cohort, with standard deviation reported in parenthesis. Bold indicates the highest average metric value across the metric for each test dataset. Differences on the ISBI test set (*n* = 5) were not statistically significant (see main text and Supplementary Table 10).

### 3.2 New lesion segmentation

New lesion segmentation performance on the clinical test set is summarised in Table 6, with additional voxel-wise metrics in Supplementary Table S11. The combined model achieved the highest numerical scores regarding lesion-wise precision and F1-score; however, no statistically significant differences were observed between any of the three models on either DSC or F1 (all *p* > 0.17, Supplementary Table S12), consistent with the substantially overlapping bootstrap 95% confidence intervals. Performance metrics for each individual subject, for all three models, are provided in Supplementary Tables S13–16.

**Table 6:** Performance of LongiSegDW with T2w FLAIR input, for new lesion segmentation when trained on different datasets.

Model variation	Train dataset	Test dataset	DSC	F1-score	Precision	Recall
LongiSegDW	Clinical (*n* = 65)	Clinical (*n* = 22)	**0.2596** **(0.143–0.383)**	0.3807 (0.218–0.548)	0.4308 (0.252–0.614)	0.3954 (0.216–0.585)
LongiSegDW	MSSEG-2 (*n* = 29)	Clinical (*n* = 22)	0.2355 (0.141–0.336)	0.4137 (0.260–0.571)	0.3563 (0.216–0.506)	**0.5535** **(0.369–0.737)**
LongiSegDW	Clinical + MSSEG-2 (*n* = 94)	Clinical (*n* = 22)	0.2535 (0.146–0.368)	**0.4191** **(0.255–0.590)**	**0.4750** **(0.287–0.661)**	0.4462 (0.226–0.631)
LongiSegDW	Clinical (*n* = 65)	MSSEG-2 (*n* = 29)	0.2743 (0.168–0.385)	0.4651 (0.331–0.601)	0.7069 (0.543–0.862)	0.3945 (0.264–0.530)

Values are means across subjects in each test cohort, with 95% bootstrap confidence intervals in parentheses (10,000 iterations, sampling with replacement). Bold indicates the highest average metric value across each metric—see main text and Supplementary Tables for pairwise significance testing. DSC, dice similarity coefficient; DW: difference weighting.

Patient-wise classification metrics for all three models on the combined cohort of active (*n* = 22) and stable (*n* = 58) patients are provided in Supplementary Table S17. Across models, recall was the strongest metric (0.68–0.91), indicating reasonable sensitivity for detecting patients with new lesions. NPV was consistently high (0.84–0.91), suggesting that a stable prediction is reliable. Precision was low across all models (0.34–0.46), reflecting a tendency to over-predict disease activity. The combined training dataset achieved the best overall balance, with the highest F1-score and balanced accuracy.

**Figure 4:**
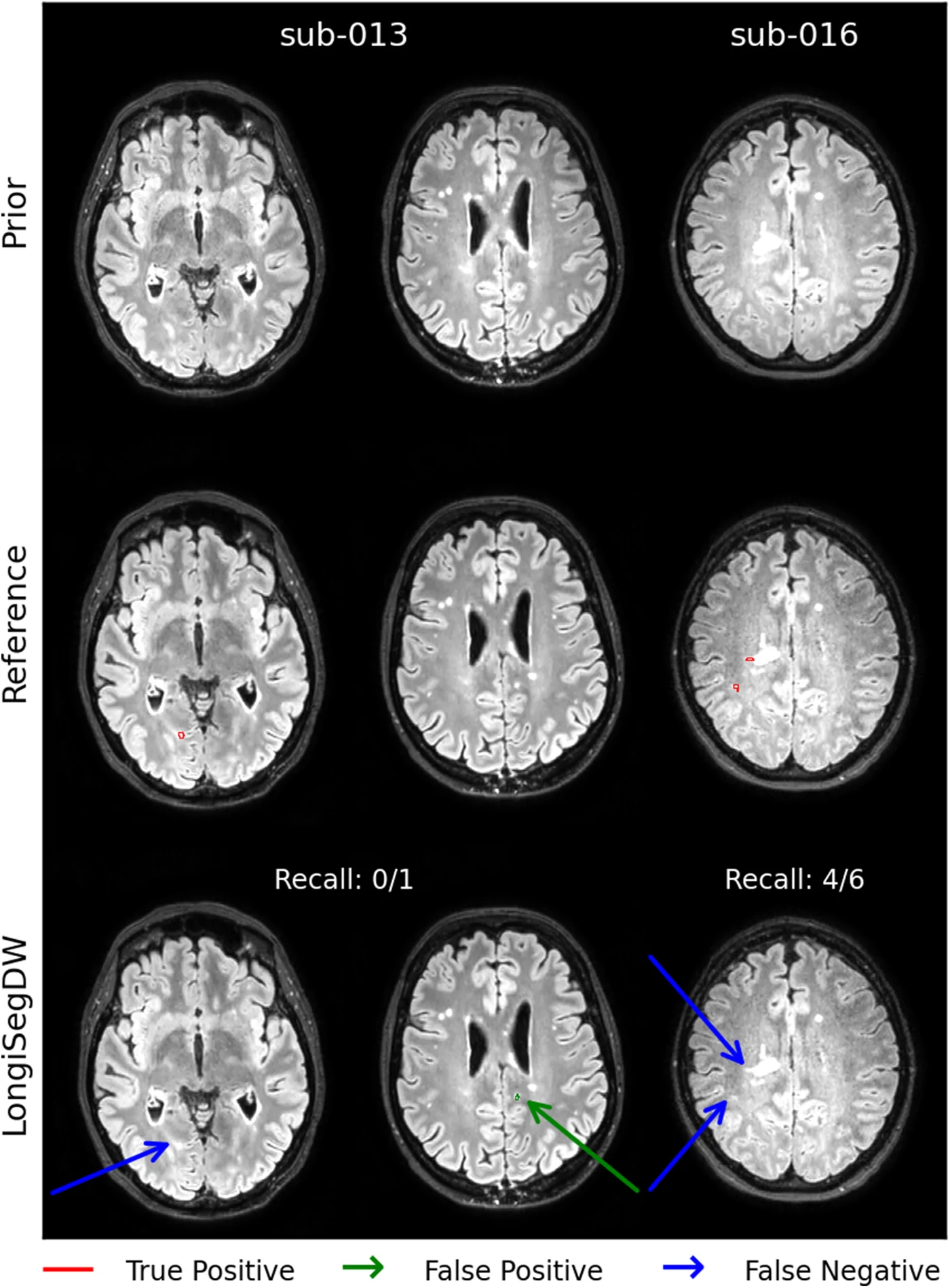
Examples of false positives and false negative delineations of new lesions in two patients from the MSSEG-2 dataset. The false positive new lesion has a vague outline of a lesion in the prior scan and should therefore not be classified as new. LongiSegDW is the LongiSeg model with difference weighting (DW), trained on the clinical new lesion train dataset (*n* = 65).

For benchmark reference, the model trained on the clinical dataset obtained an average DSC of 0.274 and F1-score of 0.465 when evaluated on the MSSEG-2 dataset (Table 6). Performance varied substantially between patients, with some obtaining a DSC around 0.78, and others near or at 0. The full list of metrics per patient is provided in Supplementary Table S14. Figure 4 illustrates examples of delineations of two patients, showcasing delineation challenges of false negative and false positive delineations.

## 4 Discussion

In this study, we trained LongiSeg—a state-of-the-art longitudinal model for cross-sectional MS lesion segmentation—on a large and heterogeneous clinical dataset to evaluate its performance. We found that LongiSeg, using T2w FLAIR MRI from two timepoints, obtained a higher segmentation accuracy compared to a model trained on single-timepoint data only. The addition of difference weighting blocks to LongiSeg (called LongiSegDW) did not provide a significant increase in performance. When both T2w FLAIR and T2w images were provided as input, LongiSeg performed significantly better than the single-timepoint model with regards to F1-score, but not DSC. As a novel contribution, we trained LongiSegDW for new lesion segmentation specifically. However, LongiSegDW did not achieve a model performance comparable with that of literature for new lesion segmentation, highlighting the challenges of this task when using real-world clinical data (26, 30, 35).

LongiSegDW improved the lesion delineation accuracy compared to single-timepoint nnU-Net (DSC 0.678 vs. 0.665). This result is consistent with the findings of Rokuss et al. (36) who reported an increase in DSC from 0.742 to 0.756 using the Ljubljana test dataset. Despite the added variability inherent to routine clinical data, our clinical model's performance approached that reported by Rokuss et al. on the more standardized, single-site Ljubljana dataset. Within our own clinical test set, we additionally did not observe a significant effect of T2w FLAIR acquisition pairing on performance (23% of cases with mixed 2D/3D acquisitions vs. 2D/2D), in contrast to what has been previously reported (49).

When training and evaluating LongiSegDW for cross-sectional lesion segmentation on the Ljubljana dataset, we achieved a DSC of 0.757 and an F1-score of 0.813. These results closely replicate those reported by Rokuss et al. (36), who obtained a DSC of 0.756 and an F1-score of 0.733. The discrepancy in F1-score is likely due to differences in data splitting strategies and evaluation protocols: Rokuss et al. averaged performance across both prior and current scans, whereas we report results based solely on current scans. In comparison, our clinical model performed significantly worse on the Ljubljana dataset. This difference can be attributed to the fact that both training and test sets originated from the same source (Ljubljana), potentially limiting generalisability. To further explore this, we evaluated both models on the ISBI dataset, which was not included in any training set. Here, the DSC and F1 scores were similar between the Ljubljana-trained and clinical models, with no significant difference detected. However, with only 5 patients in the ISBI test set, the confidence intervals around these estimates are wide, and this comparison should be considered exploratory. For reference, our Ljubljana-trained model also reproduced the ISBI results reported by Rokuss et al. (DSC: 0.708, F1-score: 0.625).

Performance was low when training LongiSegDW for new-lesion segmentation on the clinical dataset, producing segmentations in only 12 of 22 cases. This may partly reflect the substantial reduction in training data compared to cross-sectional segmentation, since only the new-lesion sub-cohort (*n* = 65) was available for training. Furthermore, this subset remained highly heterogeneous—acquired across ten scanner models, with 33% of cases showing a change in dimensionality between the prior and current scan—despite the smaller sample size, further complicating the task. In the test dataset, eight of 22 patients had only a single new lesion. When the clinical LongiSegDW model was applied to the unseen MSSEG-2 dataset, performance was similarly limited: the model failed to segment lesions in eight of 29 cases, all but one of which had only a single new lesion. This pattern, consistent across both test sets, suggests that the model struggles specifically with cases presenting few lesions. Taken together, these results indicate that the model's limited new-lesion segmentation performance likely stems from the combination of a small, heterogeneous training set and test cases with minimal lesion burden. We additionally observed no detection of new lesions when using multi-class labels, possibly because the model preferentially optimizes for the (more prevalent) stationary-lesion class.

Segmenting new MS lesions is considerably more challenging than cross-sectional lesion segmentation, as reflected by consistently lower scores in public benchmarks (50, 51). The difficulty of this task is further underscored by the substantial inter-rater variability as seen in the MSSEG-2 challenge, in which four human experts delineated the reference dataset. This resulted in inter-rater DSC values ranging from 0.461 to 0.631 and F1-scores from 0.524 to 0.712 (35).

Martinez-Heras et al. (32) presented a longitudinal model for new lesion segmentation based on the standard nnU-Net. Their model accepted two-channel input of consecutive T2w FLAIR MRI—like our clinical LongiSeg model without DW. They used the MSSEG-2 training dataset, as well as an additional 20 cases from MS Open Data (52). When testing on 12 patients from the MSSEG-2 training dataset, they got an average DSC of 0.58 and recall of 0.62. Notably, their evaluation protocol assigned perfect scores (1.0) to patients without new lesions, assuming no false positives, which inflates the average metrics. In comparison, we achieved an average DSC of 0.27 on the MSSEG-2 dataset when excluding patients without new lesions. If we were to use the same, inflated evaluation strategy, our model would achieve a DSC of 0.47 and recall of 0.58. Basaran et al. (33) also employed nnU-Net for longitudinal, new lesion segmentation, but enhanced their approach with extensive data augmentation, including synthetic lesion generation to increase dataset diversity. Their model was trained and tested on the official MSSEG-2 challenge datasets (*n* = 40/60), achieving an average DSC of 0.51 and F1-score of 0.55. The improved performance likely stems from the use of advanced augmentation techniques and synthetic data, which enriched the training set and improved generalisation. However, Basaran et al. evaluated on the official held-out MSSEG-2 test set, whereas our clinical model was trained and tested on splits drawn from the publicly available 40-case training dataset alone, limiting direct comparability.

Kamraoui et al. (24) also used a 3D U-Net-based architecture and addressed the same core problem of incorporating information from two timepoints, though via a different mechanism: a siamese encoder that extracts and aggregates features from both timepoints, compared to our difference-weighting blocks. To address the same data scarcity we encountered, they pretrained their encoder on single-timepoint segmentation and generated synthetic longitudinal pairs to pretrain their decoder, before fine-tuning on the same MSSEG-2 training cohort we use for benchmarking. This pipeline achieved an Avg. Score of 0.523 and F1 of 0.550 on the official held-out MSSEG-2 test set, using a five-model ensemble at inference. For reference, our clinical model achieved a DSC of 0.274 and F1-score of 0.465 when evaluated on MSSEG-2 (Table 6); however, differences in evaluation split (official held-out test set vs. our own split of the public training data) and inference strategy (five-model ensemble vs. single model) limit direct comparability.

The calculation of patient-wise classification metrics highlighted the current limitations of clinical applicability. Across all models, recall was the strongest metric (0.68–0.91), suggesting reasonable sensitivity for identifying patients with new lesions, which is a critical direction of error in MS monitoring, where missing disease activity has direct treatment implications. NPV was consistently high (0.84–0.91), indicating that a stable prediction is reliable. However, precision was low across all models (0.34–0.46), meaning a substantial proportion of patients flagged as active were false positives. Taken together, these results suggest that, in its current form, the model may support ruling out disease activity but would not yet reliably confirm it for clinical decision-making.

### Limitations

Only 74 of 537 patients in our primary clinical dataset exhibited one or more new lesions between the prior and current MRI examinations, resulting in a small cohort for both training and testing. This likely contributed to the limited performance of our new lesion segmentation models. The fact that such a small proportion of patients with new lesions could be identified from what is, by clinical MS imaging standards, a relatively large dataset, underlines a fundamental challenge in this domain: most patients do not develop new lesions within a given inter-scan interval, making large, annotated datasets for new lesion segmentation inherently difficult to assemble. Potential strategies to address this data scarcity include synthetic lesion generation to augment training data, as demonstrated by Kamraoui et al. (24), who generated synthetic longitudinal pairs from single-timepoint scans to pretrain their model. Federated learning approaches, which aggregate data across multiple clinical sites without centralising patient data, offer another potential direction (53, 54).

Two limitations concern the reference masks used for model training and evaluation. the new lesion reference masks in the training dataset were not validated by a trained specialist, in contrast the cross-sectional masks which underwent expert review as part of a previous study (21). This introduces greater uncertainty in the ground truth labels for new lesion segmentation and may have contributed to the limited training signal available to the model. Second, the cross-sectional reference masks were originally delineated using only the current MRI examination, without access to the prior scan. As these masks were subsequently used to train and evaluate a longitudinal model that jointly processes both timepoints, the reference standard may not fully capture lesion characteristics that would only be apparent through direct comparison with the prior examination, potentially underestimating the true lesion burden in some cases. Ideally, longitudinal reference masks would be generated with access to both timepoints, more closely reflecting the clinical decision-making process.

The selection of the best model checkpoint based on the internal nnU-Net validation loss, rather than the final checkpoint, introduces a potential risk of overfitting to the internal validation split. While this validation set is drawn exclusively from the training data and is entirely separate from the held-out test set, repeated implicit optimisation toward it across 1,000 epochs may bias the selected checkpoint. The choice was made specifically with the regards to the new lesion sub-cohort, to decrease the risk of overfitting, which could be introduced when training for 1,000 epochs on a dataset of 65 cases. The choice was made for all models for consistency.

All models were trained using a single predefined train/test split rather than a full five-fold ensemble, which is known to improve robustness and generalisation (47). Given the substantial number of models trained across two tasks, multiple dataset configurations, and different input modalities, five-fold ensemble training was not computationally feasible within the scope of this work. This may limit the robustness of individual model estimates, and performance could potentially improve with ensemble training. In particular, our cross-dataset comparisons between models trained on Ljubljana and clinical data rely on single train/test splits rather than repeated resampling and should be interpreted as preliminary evidence rather than a definitive characterisation of cross-site robustness. Similarly, benchmarking on the ISBI dataset was based on only 5 patients, limiting the statistical power of this comparison.

## 5 Conclusion

This study evaluated LongiSeg, a longitudinal deep learning framework, on a heterogeneous clinical MS dataset and attempted a novel adaptation of the network to specifically new lesion segmentation. The longitudinal model demonstrated significant improvements in cross-sectional lesion segmentation compared to single-timepoint approaches. However, the added complexity of processing consecutive scans may not be justified by the modest performance gains over simpler single-timepoint methods, especially in resource-constrained environments.

End-to-end new lesion segmentation using LongiSeg revealed substantial limitations. The model failed to produce segmentations in 45% of cases from the clinical test dataset, indicating the need for significantly larger and more balanced annotated datasets to reach clinical utility.

Until such datasets are available, hybrid approaches combining cross-sectional segmentation with *post-hoc* lesion tracking may offer a more practical solution for clinical deployment.

## Data Availability

The datasets presented in this article are not readily available because of ethical and privacy restrictions on clinical data. Data may be made available upon reasonable request and subject to approval by the relevant institutional and ethical authorities. Requests to access the datasets should be directed to Amalie Monberg Hindsholm, amalie.monberg.hindsholm@regionh.dk.

## Footnotes

^1^https://github.com/CAAI/AIMS
