# Supplementary material for: Longitudinal deep learning-based segmentation of multiple sclerosis lesions in a local clinical cohort
Source: Front Radiol. 2026 Aug 12;6:1758276. doi: 10.3389/fradi.2026.1758276 (PMC13506831; doi:10.3389/fradi.2026.1758276)
Supplement: Supplementary file 1 [file Supplementaryfile1.docx]

Supplementary Material

# 1 Supplementary Data

**Supplementary Data 1: Demographic Information, Clinical Dataset**

Demographics and acquisition parameters: The median age at the follow-up scan was 45 years (range [20,76]), with 70% female. Images were acquired using 15 different scanner types, including both 1.5T and 3T models, distributed across three main vendors: GE (GE Healthcare, Illinois, USA) 17%, Siemens (Siemens Healthineers, Erlangen, Germany) 61%, and Philips (Philips Healthcare, Best, The Netherlands) 22%. Protocols were not standardized between scanners, but all included a T2-weighted (T2w) fluid attenuated inversion recovery (T2w FLAIR) sequence, in either 3D or 2D (50%/50%), and a 2D T2w sequence. In 38% of cases, both T2w FLAIR scans from a patient were 2D, in 39% both were 3D, and in 23% the prior and follow-up T2w FLAIR scans were acquired using different MR acquisition dimensionalities.

**Supplementary Data 2: Acquisition Details, Public Datasets**

Ljubljana: Acquisition parameters: Each time point contains a 3D T2w FLAIR and a 2D T1w turbo inversion recovery magnitude (TIRM) image, acquired on a 3T Siemens Magnetom Trio MR system (Siemens Healthcare, Erlangen, Germany).

ISBI: Acquisition parameters: All scans were acquired on a 3T MRI scanner (Philips Medical Systems, Best, The Netherlands).

MSSEG-2: Acquisition parameters: Only 3D T2w FLAIR images were included in this dataset. The images were acquired at one of 15 different MR scanners, distributed across two of the main vendors: Siemens (Siemens AG, München, Germany), and Philips (Philips Healthcare, Best, The Netherlands). The models included both 1.5T and 3T scanners.

# 2 Supplementary Figures and Tables

## 2.1 Supplementary Tables

**Supplementary Table 1:** Acquisition Parameters from “prior” scans

|  | **Signa HDxt** | **Avanto** | **Achieva dStream** | **Trio** | **Verio** | **Prisma_fit** | **SIGNA HDe** | **Optima MR450w** | **Avanto_fit** | **Achieva** | **Aera** | **DISCOVERY MR450** | **Optima_ MR450w** | **Symphony** | **Panorama HFO** | **Ingenia** |
| --- | --- | --- | --- | --- | --- | --- | --- | --- | --- | --- | --- | --- | --- | --- | --- | --- |
| **N** | 23 | 149 | 102 | 45 | 105 | 45 | 12 | 48 | 2 | 10 | 2 | 1 | 1 | 1 | 3 | 1 |
| **MRAcquisition** | 2D | 3D | 2D | 2D | 3D | 3D | 2D | 2D | 3D | 2D | 2D | 3D | 2D | 2D | 2D | 3D |
| **Echo Time [ms]** | 138.6 | 333.0 | 125.0 | 97.0 | 395.0 | 387.0 | 86.4 | 91.6 | 335.0 | 125.0 | 89.0 | 103.9 | 91.6 | 115.0 | 140.0 | 295.7 |
| **Repetition Time [ms]** | 8002.0 | 5000.0 | 11000.0 | 10000.0 | 5000.0 | 5000.0 | 8002.0 | 9000.0 | 5000.0 | 11000.0 | 5000.0 | 5000.0 | 9000.0 | 8150.0 | 11000.0 | 4800.0 |
| **Inversion Time [ms]** | 2000.0 | 1800.0 | 2800.0 | 2500.0 | 1800.0 | 1800.0 | 2000.0 | 2579.1 | 1800.0 | 2800.0 | 1800.0 | 1672.0 | 2579.1 | 2500.0 | 2800.0 | 1650.0 |
| **Flip Angle** | 90 | 120 | 90 | 150 | 120 | 120 | 90 | 160 | 120 | 90 | 120 | 90 | 160 | 150 | 90 | 90 |
| **Slice Thickness [mm]** | 4.0 | 1.0 | 3.5 | 4.0 | 1.0 | 0.9 | 4.0 | 4.0 | 0.7 | 3.5 | 1.0 | 1.2 | 4.0 | 5.0 | 3.0 | 1.1 |

**Supplementary Table 2**: Acquisition Parameters from “current” scans

|  | **Achieva dStream** | **Trio** | **Optima MR450w** | **Prisma_fit** | **Verio** | **Avanto** | **Signa HDxt** |
| --- | --- | --- | --- | --- | --- | --- | --- |
| **N** | 104 | 89 | 82 | 84 | 78 | 80 | 33 |
| **MRAcquisition** | 2D | 2D | 2D | 3D | 3D | 3D | 3D |
| **Echo Time [ms]** | 125.0 | 97.0 | 97.6 | 388.0 | 395.0 | 333.0 | 124.1 |
| **Repetition Time [ms]** | 11000.0 | 10000.0 | 9000.0 | 5000.0 | 5000.0 | 5000.0 | 6000.0 |
| **Inversion Time [ms]** | 2800.0 | 2500.0 | 2579.5 | 1800.0 | 1800.0 | 1800.0 | 2000.0 |
| **Flip Angle** | 90 | 150 | 160 | 120 | 120 | 120 | 90 |
| **Slice Thickness [mm]** | 3.5 | 4.0 | 4.0 | 1.0 | 1.0 | 1.0 | 1.8 |

**Supplementary Table 3:** Key configuration parameters for each of the trained models.

| **Dataset** | Clinical | Clinical | Ljubljana | Clinical, new lesion cohort* | MSSEG-2** | Combined * and ** |
| --- | --- | --- | --- | --- | --- | --- |
| **Model** | nnU-Net | LongiSeg/DW | LongiSeg/DW | LongiSeg/DW | LongiSeg/DW | LongiSeg/DW |
| **Input voxel spacing (mm)** | 0.72×0.60×0.72 | 0.72×0.60×0.72 | 1.0×1.0×1.0 | 0.72×0.60×0.72 | 0.98×0.98×0.53 | 1.02×0.60×0.72 |
| **Patch size (voxels)** | 192x256x160 | 192x256x160 | 160x192x160 | 160x288x160 | 160x192x256 | 128x288x192 |
| **Median shape after preprocessing (voxels)** | 189x281x185 | 189x281x185 | 131×171×137 | 187×283×184 | 136×170×251 | 133×280×184 |
| **Batch size** | 2 | 2 | 3 | 2 | 2 | 2 |
| **Preprocessor** | Default | LongiSeg | LongiSeg | LongiSeg | LongiSeg | LongiSeg |
| **Normalisation** | ZScore | ZScore | ZScore$\dagger$ | ZScore | ZScore | ZScore |

$\dagger$Ljubljana model uses two ZScore normalisation channels, one per input modality (T1w and T2w FLAIR).

**Supplementary Table 4:** Shared network configuration across all trained models.

| **Parameter** | **Value** |
| --- | --- |
| Network | 3d_fullres |
| Trainer | nnUNetTrainer |
| Initial learning rate | 0.01 |
| Number of base features | 32 |
| Features per stage | [32, 64, 128, 256, 320, 320] |
| Number of stages | 6 |
| Normalisation layer | InstanceNorm3d |
| Activation | LeakyReLU |
| Max epochs | 1000 |
| Batches per epoch | 250 |
| Net_conv_kernel_sizes | [[3,3,3] x 6 stages] |
| Net_num_pool_op_kernel_sizes | [[2,2,2] x 5 stages] |
| Net_pool_per_axis | [5,5,5] |
| Plan | nnUNetResEncUNetLPlans |
| Framework version | NnU-Net v2 |

**Supplementary Table 5:** Voxel-wise segmentation metrics for cross-sectional lesion segmentation models evaluated on the clinical test set (n=67). Voxel-wise F1-score, precision, and recall are reported as mean (standard deviation). The Pearson correlation coefficient (r) was computed between predicted and reference total lesion volumes per patient. All Pearson correlations were statistically significant (p<0.001).

| **Model Name** | **Train dataset** | **Voxel-wise F1-score** | **Voxel-wise Precision** | **Voxel-wise Recall** | **Pearson-coefficient** |
| --- | --- | --- | --- | --- | --- |
| **Models trained with T2w FLAIR input** | | | | | |
| nnU-Net | clinical, single timepoint | 0.6648 (0.1438) | 0.814 (0.1382) | 0.5913 (0.1774) | r=0.9641, p<0.001 |
| LongiSeg | clinical, longitudinal | 0.6838 (0.1243) | 0.7888 (0.1256) | 0.6286 (0.1641) | r=0.9656, p<0-001 |
| LongiSegDW | clinical, longitudinal | 0.6782 (0.1287) | 0.7909 (0.1208) | 0.6204 (0.1679) | r=0.9661, p<0.001 |
| **Models trained with T2w FLAIR and T2w input** | | | | | |
| nnU-Net | clinical, single timepoint | 0.6695 (0.1368) | 0.8040 (0.1322) | 0.6020 (0.1743) | r=0.9583, p<0.001 |
| LongiSegDW | clinical, longitudinal | 0.5662 (0.1342) | 0.6996 (0.1503) | 0.5081 (0.1744) | r=0.8928, p<0.001 |

**Supplementary Table 6:** Pairwise Wilcoxon signed-rank test results for cross-sectional lesion segmentation — T2w FLAIR-only models (n=67). Bonferroni-corrected threshold p<0.017. Positive median difference indicates the second model outperforms the first.

| **Comparison** | **DSC median difference** | **DSC p-value** | **F1 median difference** | **F1 p-value** |
| --- | --- | --- | --- | --- |
| nnU-Net vs LongiSeg | +0.014 | <0.001* | +0.018 | <0.001* |
| nnU-Net vs LongiSegDW | +0.009 | <0.001* | +0.016 | 0.002* |
| LongiSeg vs LongiSegDW | -0.005 | 0.009 | +0.000 | 0.156 |

**Supplementary Table 7:** Pairwise Wilcoxon signed-rank test results for cross-sectional lesion segmentation, testing the effect of one (T2w FLAIR)- vs two-channel (T2w FLAIR+T2w) input across model types (n=67). No Bonferroni correction is applied as each comparison addresses an independent hypothesis. Positive median difference indicates the second model outperforms the first.

| **Comparison** | **DSC median difference** | **DSC p-value** | **F1 median difference** | **F1 p-value** |
| --- | --- | --- | --- | --- |
| nnU-Net with T2w vs LongiSegDW with T2w | +0.003 | 0.131 | +0.024 | 0.039 |
| LongiSegDW vs LongiSegDW with T2w | -0.000 | 0.950 | +0.015 | 0.191 |
| nnU-Net vs nnU-Net with T2w | +0.002 | 0.110 | +0.002 | 0.125 |

**Supplementary Table 8**: Pairwise Mann-Whitney U-tests comparing LongiSegDW performance across T2w FLAIR acquisition pairing subgroups on the clinical test set (2D→2D, n=25; 3D→3D, n=24; 2D→3D, n=18). U-statistics and two-sided p-values are reported for each metric. Bonferroni correction was applied to account for multiple comparisons within each metric (three pairwise comparisons per metric, corrected significance threshold p<0.017). None of the comparisons survived Bonferroni correction. DSC: Dice Similarity Coefficient.

| **Comparison** | **DSC** | **F1-score** | **Precision** | **Recall** |
| --- | --- | --- | --- | --- |
| 2D,2D vs 3D,3D | U=374, p=0.1416 | U=328, p=0.5755 | U=142, p=0.0015 | U=393, p=0.0642 |
| 2D,2D vs 2D,3D | U=253, p=0.4984 | U=300, p=0.0648 | U=168, p=0.1633 | U=316, p=0.0259 |
| 3D,3D vs 2D,3D | U=182, p=0.3945 | U=268, p=0.1949 | U=296, p=0.0402 | U=251, p=0.3804 |

**Supplementary Table 9:** Voxel-wise segmentation metrics for LongiSegDW evaluated on public benchmark datasets. Voxel-wise F1-score, precision, and recall are reported as mean (standard deviation). The Pearson correlation coefficient (r) was computed between predicted and reference total lesion volumes per patient. All Pearson correlations were statistically significant (p<0.001).

| Model (input) | Train dataset | Test dataset | Voxel-wise F1-score | Voxel-wise Precision | Voxel-wise Recall | Pearson-coefficient |
| --- | --- | --- | --- | --- | --- | --- |
| LongiSegDW (T2w FLAIR & T1w) | Ljubljana (n=129) | Ljubljana (n=33) | 0.7568 (0.0806) | 0.7836 (0.0704) | 0.7417 (0.1099) | r=0.9945, p<0.001 |
| LongiSegDW  (T2w FLAIR) | clinical (n=472) | Ljubljana (n=33) | 0.6654 (0.1128) | 0.7614 (0.0963) | 0.6184 (0.1554) | r=0.9791, p<0.001 |
| LongiSegDW   (T2w FLAIR & T1w) | Ljubljana (n=129) | ISBI (n=5) | 0.7223 (0.0528) | 0.8940 (0.0258) | 0.6089 (0.0710) | r=0.9992, p<0.001 |
| LongiSegDW  (T2w FLAIR) | clinical | ISBI (n=5) | 0.7242 (0.0542) | 0.8426 (0.0492) | 0.6497 (0.0762) | r=0.9960, p<0.001 |

**Supplementary Table 10**: Pairwise Wilcoxon signed-rank test results for benchmark segmentation. No Bonferroni correction applied as there is one comparison per test set. Positive median difference indicates the clinical model outperforms the Ljubljana model. ISBI results should be interpreted with caution due to the small sample size (n=5).

| **Comparison** | **n** | **DSC median difference** | **DSC p-value** | **F1 median difference** | **F1 p-value** |
| --- | --- | --- | --- | --- | --- |
| Ljubljana vs Clinical (Ljubljana test set) | 33 | −0.073 | <0.001* | −0.051 | <0.001* |
| Ljubljana vs Clinical (ISBI test set) | 5 | −0.001 | 1.000 | -0.037 | 0.625 |

Supplementary Table 11: Voxel-wise segmentation metrics for new lesion segmentation models evaluated on clinical and public test sets. Voxel-wise F1-score, precision, and recall are reported as mean (standard deviation). The Pearson correlation coefficient (r) was computed between predicted and reference total lesion volumes per patient, with corresponding p-values reported.

| Model variation | Train dataset | Test dataset | Voxel-wise F1-score | Voxel-wise Precision | Voxel-wise Recall | Pearson-coefficient |
| --- | --- | --- | --- | --- | --- | --- |
| LongiSegDW | clinical (n=65) | clinical (n=22) | 0.2596 (0.2916) | 0.7740 (0.3388) | 0.2405 (0.2914) | r=0.2019, p=0.3675 |
| LongiSegDW | MSSEG-2 (n=29) | clinical (n=22) | 0.2355 (0.2344) | 0.4700 (0.4059) | 0.2775 (0.2751) | r=0.6814, p=0.0005 |
| LongiSegDW | Clinical + MSSEG-2 (n=94) | clinical (n=22) | 0.2535 (0.2664) | 0.8544 (0.2684) | 0.2350 (0.2652) | r=0.1486, p=0.5092 |
| LongiSegDW | clinical (n=65) | MSSEG-2 (n=29) | 0.2743 (0.3015) | 0.9430 (0.1866) | 0.2026 (0.2411) | r=0.5707, p=0.0012 |

**Supplementary Table 12:** Pairwise Wilcoxon signed-rank test results for new lesion segmentation (n=22). Bonferroni-corrected threshold p<0.017. No statistically significant differences were observed between models for new lesion segmentation. Positive median difference indicates the second model outperforms the first.

| **Comparison** | **DSC median difference** | **DSC p-value** | **F1 median difference** | **F1 p-value** |
| --- | --- | --- | --- | --- |
| Clinical vs MSSEG-2 | −0.0051 | 0.427 | 0.000 | 0.533 |
| Clinical vs Combined | 0.000 | 0.422 | 0.000 | 0.249 |
| MSSEG-2 vs Combined | 0.000 | 0.510 | 0.000 | 0.646 |

**Supplementary Table 13:** Individual patient performance metrics of the LongiSegDW model for new lesion segmentation trained and tested on the clinical dataset (n = 65/22).

| **Subject ID** | **DSC** | **F1-score** | **Precision** | **Recall** | **No of true positive lesions** |
| --- | --- | --- | --- | --- | --- |
| sub-01 | 0.0 | 0 | 0 | 0.0 | 0 |
| sub-02 | 0.5797 | 0.6667 | 0.5 | 1.0 | 1 |
| sub-03 | 0.6929 | 1.0 | 1.0 | 1.0 | 1 |
| sub-04 | 0.0 | 0 | 0 | 0.0 | 0 |
| sub-05 | 0.1928 | 0.2857 | 0.5 | 0.2 | 1 |
| sub-06 | 0.0 | 0 | 0 | 0.0 | 0 |
| sub-07 | 0.0 | 0 | 0 | 0.0 | 0 |
| sub-08 | 0.0 | 0 | 0 | 0.0 | 0 |
| sub-09 | 0.7613 | 0.8571 | 0.75 | 1.0 | 3 |
| sub-10 | 0.0 | 0 | 0 | 0.0 | 0 |
| sub-11 | 0.4969 | 1.0 | 1.0 | 1.0 | 1 |
| sub-12 | 0.2113 | 0.3333 | 0.5 | 0.2500 | 1 |
| sub-13 | 0.4242 | 0.6667 | 0.8 | 0.5714 | 4 |
| sub-14 | 0.0 | 0 | 0 | 0.0 | 0 |
| sub-15 | 0.6296 | 1.0 | 1.0 | 1.0 | 1 |
| sub-16 | 0.2199 | 0.6 | 0.4286 | 1.0 | 3 |
| sub-17 | 0.5961 | 0.6667 | 1.0 | 0.5 | 1 |
| sub-18 | 0.7977 | 1.0 | 1.0 | 1.0 | 1 |
| sub-19 | 0.0 | 0 | 0 | 0.0 | 0 |
| sub-20 | 0.0 | 0 | 0 | 0.0 | 0 |
| sub-21 | 0.1078 | 0.3 | 1.0 | 0.1765 | 3 |
| sub-22 | 0.0 | 0 | 0 | 0.0 | 0 |

**Supplementary Table 14:** Individual patient performance metrics of the LongiSegDW model for new lesion segmentation trained on the MSSEG-2 dataset and tested on the clinical dataset (n = 29/22).

| **Subject ID** | **DSC** | **F1-score** | **Precision** | **Recall** | **No of true positive lesions** |
| --- | --- | --- | --- | --- | --- |
| sub-01 | 0.3757 | 0.5 | 0.3333 | 1.0 | 1 |
| sub-02 | 0.1979 | 0.25 | 0.1429 | 1.0 | 1 |
| sub-03 | 0.6209 | 1.0 | 1.0 | 1.0 | 1 |
| sub-04 | 0.0 | 0 | 0 | 0.0 | 0 |
| sub-05 | 0.1826 | 0.2667 | 0.2 | 0.4 | 2 |
| sub-06 | 0.2581 | 0.6667 | 0.5 | 1.0 | 1 |
| sub-07 | 0.0 | 0.0 | 0.0 | 0.0 | 0 |
| sub-08 | 0.0 | 0.0 | 0.0 | 0.0 | 0 |
| sub-09 | 0.7180 | 0.8571 | 0.75 | 1.0 | 3 |
| sub-10 | 0.0 | 0 | 0.0 | 0.0 | 0 |
| sub-11 | 0.3194 | 1.0 | 1.0 | 1.0 | 1 |
| sub-12 | 0.0772 | 0.5455 | 0.4286 | 0.75 | 3 |
| sub-13 | 0.3783 | 0.7143 | 0.7143 | 0.7143 | 5 |
| sub-14 | 0.0 | 0.0 | 0 | 0.0 | 0 |
| sub-15 | 0.4476 | 1.0 | 1.0 | 1.0 | 1 |
| sub-16 | 0.0 | 0 | 0.0 | 0.0 | 0 |
| sub-17 | 0.1742 | 0.3333 | 0.25 | 0.5 | 1 |
| sub-18 | 0.2488 | 0.6637 | 0.5 | 1.0 | 1 |
| sub-19 | 0.0 | 0 | 0.0 | 0.0 | 0 |
| sub-20 | 0.0 | 0 | 0.0 | 0.0 | 0 |
| sub-21 | 0.6419 | 0.6341 | 0.52 | 0.8125 | 13 |
| sub-22 | 0.5392 | 0.6667 | 0.5 | 1.0 | 1 |

**Supplementary Table 15:** Individual patient performance metrics of the LongiSegDW model for new lesion segmentation trained on the unified MSSEG-2 and clinical dataset (n=94) and tested on the clinical test dataset (n = 22).

| **Subject ID** | **DSC** | **F1-score** | **Precision** | **Recall** | **No of true positive lesions** |
| --- | --- | --- | --- | --- | --- |
| sub-01 | 0.0 | 0 | 0.0 | 0.0 | 0 |
| sub-02 | 0.5634 | 0.6667 | 0.5 | 1.0 | 1 |
| sub-03 | 0.5923 | 1.0 | 1.0 | 1.0 | 1 |
| sub-04 | 0.0 | 0 | 0.0 | 0.0 | 0 |
| sub-05 | 0.2 | 0.3334 | 1.0 | 0.2 | 1 |
| sub-06 | 0.0 | 0 | 0.0 | 0.0 | 0 |
| sub-07 | 0.0 | 0 | 0.0 | 0.0 | 0 |
| sub-08 | 0.0 | 0.0 | 0.0 | 0.0 | 0 |
| sub-09 | 0.7345 | 0.8571 | 0.75 | 1.0 | 3 |
| sub-10 | 0.0 | 0 | 0.0 | 0.0 | 0 |
| sub-11 | 0.3194 | 1.0 | 1.0 | 1.0 | 1 |
| sub-12 | 0.2162 | 0.5714 | 0.6667 | 0.5 | 2 |
| sub-13 | 0.4753 | 0.7692 | 0.8333 | 0.7143 | 5 |
| sub-14 | 0.0 | 0 | 0.0 | 0.0 | 0 |
| sub-15 | 0.6628 | 1.0 | 1.0 | 1.0 | 1 |
| sub-16 | 0.0965 | 0.3077 | 0.2 | 0.6667 | 2 |
| sub-17 | 0.3026 | 0.6667 | 1.0 | 0.5 | 1 |
| sub-18 | 0.7197 | 1.0 | 1.0 | 1.0 | 1 |
| sub-19 | 0.0 | 0 | 0.0 | 0.0 | 0 |
| sub-20 | 0.0 | 0 | 0.0 | 0.0 | 0 |
| sub-21 | 0.1830 | 0.3810 | 1.0 | 0.2353 | 4 |
| sub-22 | 0.5118 | 0.6667 | 0.5 | 1.0 | 1 |

**Supplementary Table 16:** Individual patient performance metrics of the LongiSegDW model for new lesion segmentation trained on the clinical dataset (n = 65) and tested on the MSSEG-2 dataset (n = 29).

| **Subject ID** | **DSC** | **F1-score** | **Precision** | **Recall** | **No of true positive lesions** |
| --- | --- | --- | --- | --- | --- |
| sub-013 | 0.0 | 0.0 | 0.0 | 0.0 | 0 |
| sub-016 | 0.6535 | 0.8 | 1.0 | 0.6667 | 4 |
| sub-018 | 0.0 | 0.0 | 0.0 | 0.0 | 0 |
| sub-020 | 0.7749 | 1.0 | 1.0 | 1.0 | 1 |
| sub-021 | 0.2197 | 0.6667 | 1.0 | 0.5 | 2 |
| sub-024 | 0.0 | 0.0 | 0.0 | 0.0 | 0 |
| sub-026 | 0.0366 | 0.2 | 1.0 | 0.1111 | 1 |
| sub-027 | 0.0768 | 0.3333 | 0.5 | 0.25 | 1 |
| sub-029 | 0.0 | 0.0 | 0.0 | 0.0 | 0 |
| sub-030 | 0.6061 | 0.8 | 1.0 | 0.6667 | 2 |
| sub-032 | 0.0 | 0.0 | 0.0 | 0.0 | 0 |
| sub-035 | 0.5460 | 0.4 | 1.0 | 0.25 | 1 |
| sub-037 | 0.6875 | 1.0 | 1.0 | 1.0 | 1 |
| sub-039 | 0.0379 | 0.4 | 1.0 | 0.25 | 3 |
| sub-043 | 0.2233 | 0.4 | 1.0 | 0.25 | 1 |
| sub-047 | 0.0 | 0.0 | 0.0 | 0.0 | 0 |
| sub-048 | 0.7856 | 1.0 | 1.0 | 1.0 | 1 |
| sub-057 | 0.0225 | 0.11768 | 1.0 | 0.0625 | 1 |
| sub-061 | 0.0853 | 0.4 | 1.0 | 0.25 | 3 |
| sub-069 | 0.0385 | 0.4 | 1.0 | 0.25 | 1 |
| sub-074 | 0.2242 | 0.6154 | 1.0 | 0.4444 | 4 |
| sub-077 | 0.0749 | 1.0 | 1.0 | 1.0 | 1 |
| sub-083 | 0.7689 | 1.0 | 1.0 | 1.0 | 2 |
| sub-088 | 0.0 | 0 | 0 | 0.0 | 0 |
| sub-091 | 0.5776 | 0.8 | 1.0 | 0.6667 | 2 |
| sub-094 | 0.0 | 0 | 0 | 0.0 | 0 |
| sub-095 | 0.1668 | 0.4878 | 1.0 | 0.3226 | 10 |
| sub-099 | 0.6929 | 1.0 | 1.0 | 1.0 | 1 |
| sub-100 | 0.6558 | 0.6667 | 1.0 | 0.5 | 1 |

Supplementary Table 17: Patient-wise classification metrics for new lesion segmentation models evaluated on clinical and public test sets. Each patient was classified as active (≥1 new lesion voxel) or stable (empty mask). Metrics were computed on the combined cohort of active and stable patients (clinical test set: n=22 active, n=58 stable; MSSEG-2 test set: n=29 active). All metrics are reported as cohort-level estimates (95% bootstrap confidence interval, 10,000 resampling iterations).

| Train dataset | Test dataset | Patient-wise F1-score | Patient-wise precision | Patient-wise recall | Patient-wise specificity | Patient-wise NPV | Balanced accuracy |
| --- | --- | --- | --- | --- | --- | --- | --- |
| clinical (n=65) | clinical (n=22) | 0.5085 (0.3255-0.6567) | 0.4054 (0.2308-0.5714) | 0.6818 (0.4665-0.8750) | 0.6207 (0.4909-0.7377) | 0.8372 (0.7249-0.9444) | 0.6513 (0.5327-0.7680) |
| MSSEG-2 (n=29) | clinical (n=22) | 0.4938 (0.3428-0.6155) | 0.3390 (0.2105-0.4584) | 0.9091 (0.7778-1.0) | 0.3276 (0.2105-0.4529) | 0.9048 (0.7619-1.0) | 0.6183 (0.5333-0.7000) |
| Clinical + MSSEG-2 (n=94) | clinical (n=22) | 0.5614 (0.3749-0.7124) | 0.4571 (0.2820-0.6286) | 0.7273 0.5200-0.9091) | 0.6724 (0.5516-0.7895) | 0.8667 (0.3749-0.7124) | 0.6998 (0.5755-0.8109) |
| clinical (n=65) | MSSEG-2 (n=29) | 0.9643 (0.9020-1.0) | 1.0 (1.0-1.0) | 0.931 (0.8214-1.0) | 1.0 (1.0-1.0) | 0.8462 (0.5833-1.0) | 0.9655 (0.9107-1.0) |

## 2.2 Supplementary Figures

## 2.3 Supplementary Figure 1. Train and validation loss for nnU-Net with one-channel input of T2w FLAIR, trained on the clinical, cross-sectional dataset (n=472).

**Supplementary Figure 2.** Train and validation loss for LongiSeg with one-channel input of T2w FLAIR, trained on the clinical, cross-sectional dataset (n=472).

**Supplementary Figure 3.** Train and validation loss for LongiSegDW with one-channel input of T2w FLAIR, trained on the clinical, cross-sectional dataset (n=472).

**Supplementary Figure 4.** Train and validation loss for nnU-Net with two-channel input of T2w FLAIR and T2w sequences, trained on the clinical, cross-sectional dataset (n=472).

**Supplementary Figure 5.** Train and validation loss for LongiSegDW with two-channel input of T2w FLAIR and T2w sequences, trained on the clinical, cross-sectional dataset (n=472).

**Supplementary Figure 6.** Train and validation loss for LongiSegDW with two-channel input of T2w FLAIR and T1w sequences, trained on the public, cross-sectional dataset, Ljubljana (n=129).

**Supplementary Figure 7.** Train and validation loss for LongiSegDW with one-channel input of T2w FLAIR, trained on the clinical, new lesion dataset (n=65).

**Supplementary Figure 8.** Train and validation loss for LongiSegDW with one-channel input of T2w FLAIR, trained on the public, new lesion dataset MSSEG-2 (n=29).

**Supplementary Figure 9.** Train and validation loss for LongiSegDW with one-channel input of T2w FLAIR, trained on the combined dataset MSSEG-2 and the clinical new lesion dataset (n=94).

**Supplementary Figure 10**: Segmentation examples from all three cross-sectional models for a representative patient with a DSC close to the median LongiSegDW performance on the clinical test set (DSC=0.69). Upper row: full axial slice with delineations overlaid. Lower row: close-up view of a sub-cortical lesion. Columns show results for the reference, nnU-Net, LongiSeg, and LongiSegDW respectively.

**Supplementary Figure 11.** Distribution of DSC and F1-scores the LongiSegDW model trained and tested on the clinical dataset (n=472/67 train/test), with a single-sequence T2w FLAIR input, stratified by acquisition pairs. The low mean DSC of the 3D->3D pairs appear to be driven by three very low scores.

**Supplementary Figure 12.** The lesion load distribution across the three acquisition pairing groups.
